# Supplementary material for: The Effect of Low and High Dose Deoxynivalenol on Intestinal Morphology, Distribution, and Expression of Inflammatory Cytokines of Weaning Rabbits
Source: Toxins (Basel). 2019 Aug 13;11(8):473. doi: 10.3390/toxins11080473 (PMC6722598; doi:10.3390/toxins11080473)
Supplement: Supplementary file 1 [file toxins-11-00473-s001.pdf]

Supplementary Materials: The Effect of Low and High Dose Deoxynivalenol on Intestinal Morphology, Distribution, and Expression of Inflammatory Cytokines of Weaning Rabbits

WanYing Yang, LiBo Huang, PengWei Wang, ZhiChao Wu, FuChang Li and ChunYang Wang

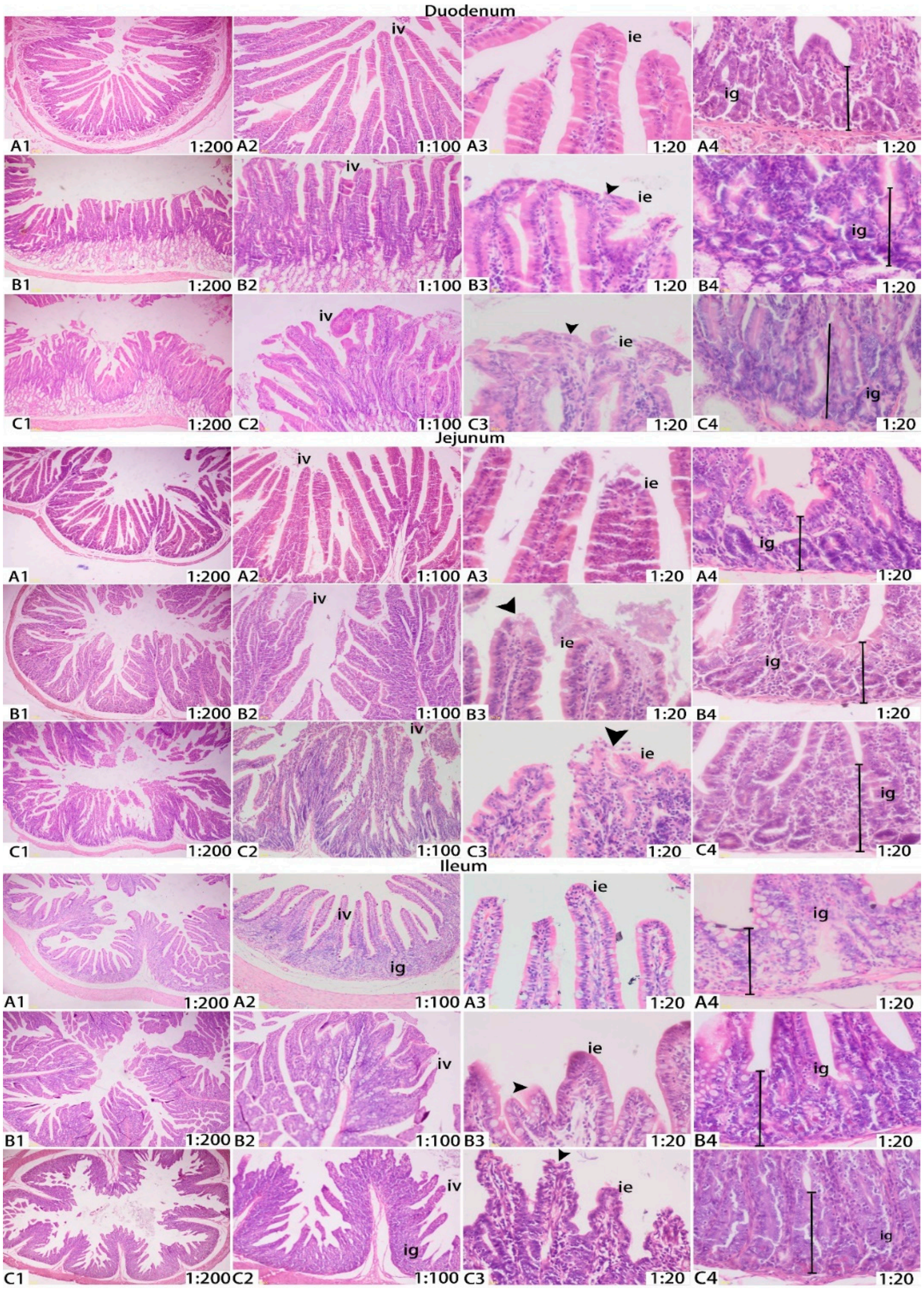

Figure S1. Effect of DON on intestinal morphology of different intestine segments by HE staining (n = 5). Group A means the control group, groups B and C means added DON at 0.5 mg/kg BW and 1.5 mg/kg BW respectively. iv means villi, ie, means intestinal epithelium and ig means intestinal gland. Scale bar was 20 μm. The 1:200, 1:100 and 1:20 represent the magnification of electron microscopy is 40×, 100× and 400× respectively.

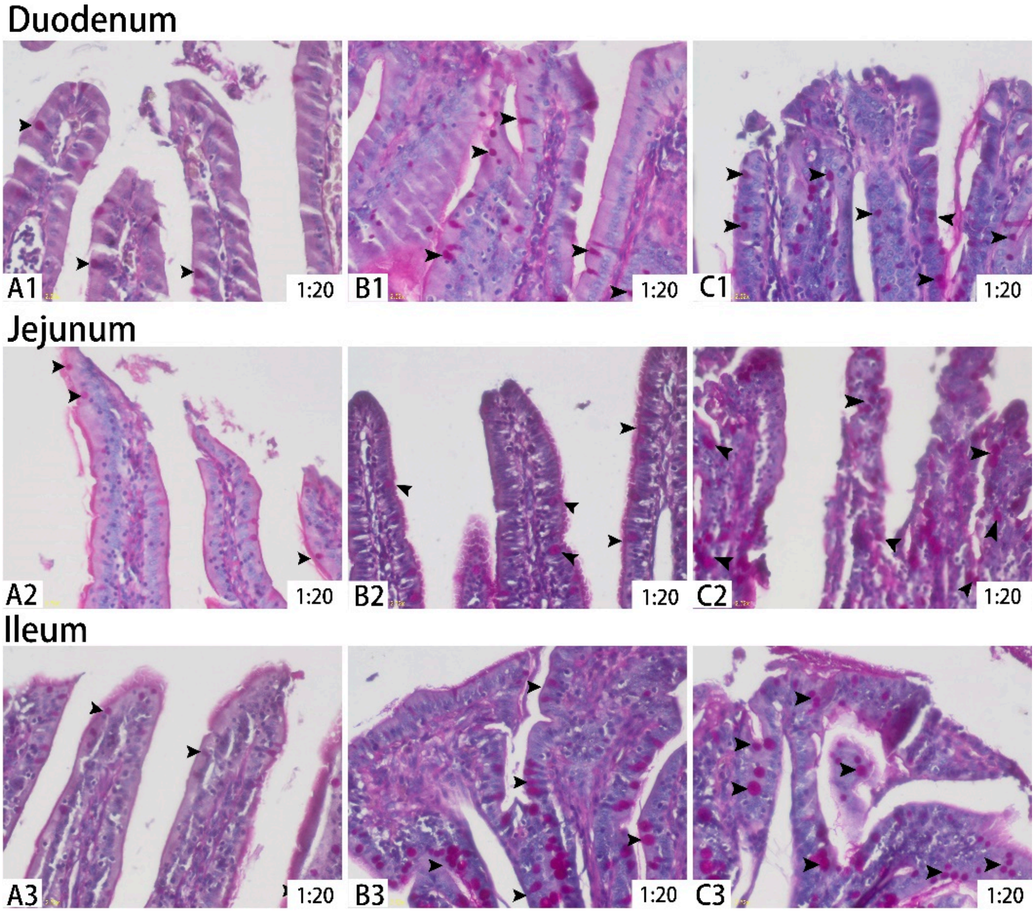

Figure S2. Effect of DON on goblet cell in different intestine segments stained by PAS. Group A means the control group, while groups B and C means added DON at 0.5 mg/kg BW and 1.5 mg/kg BW respectively. The goblet cell was emphasized by arrowhead. The 1:20 represent the magnification of electron microscopy is 400×.

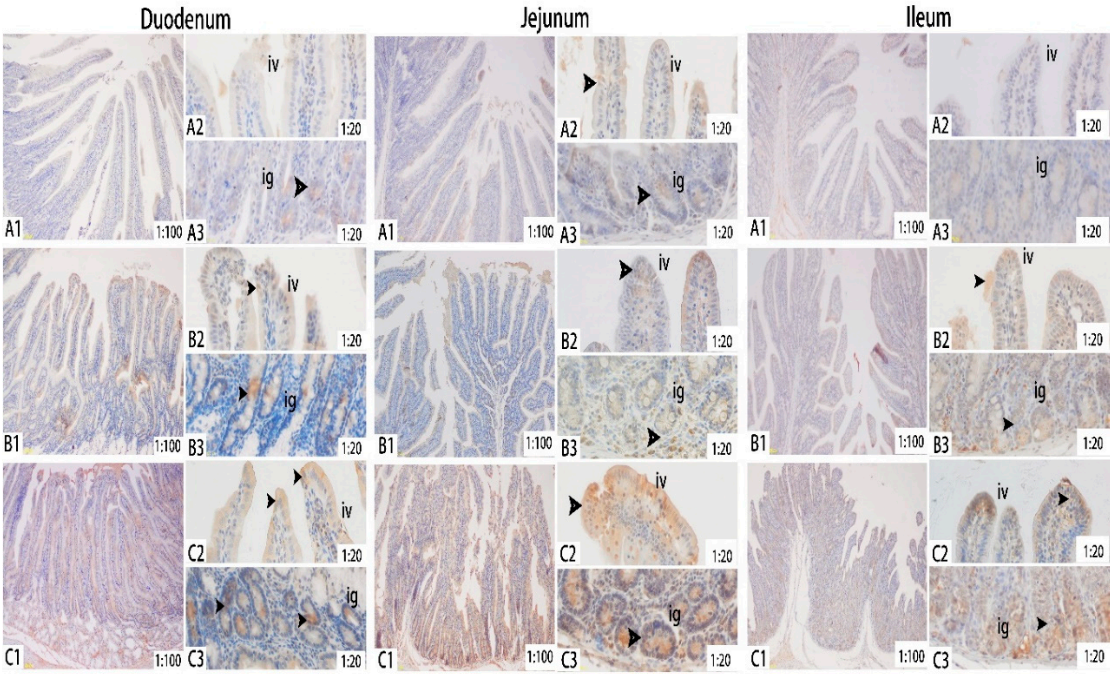

Figure S3. The distribution of IL-1β was tested via IHC method. Group A means the control group, groups B and C means added DON at 0.5 mg/kg BW and 1.5 mg/kg BW respectively. The 1:100 and

1:20 represent the magnification of electron microscopy is 100× and 400× respectively. The brown positive reactants was emphasized by arrow.

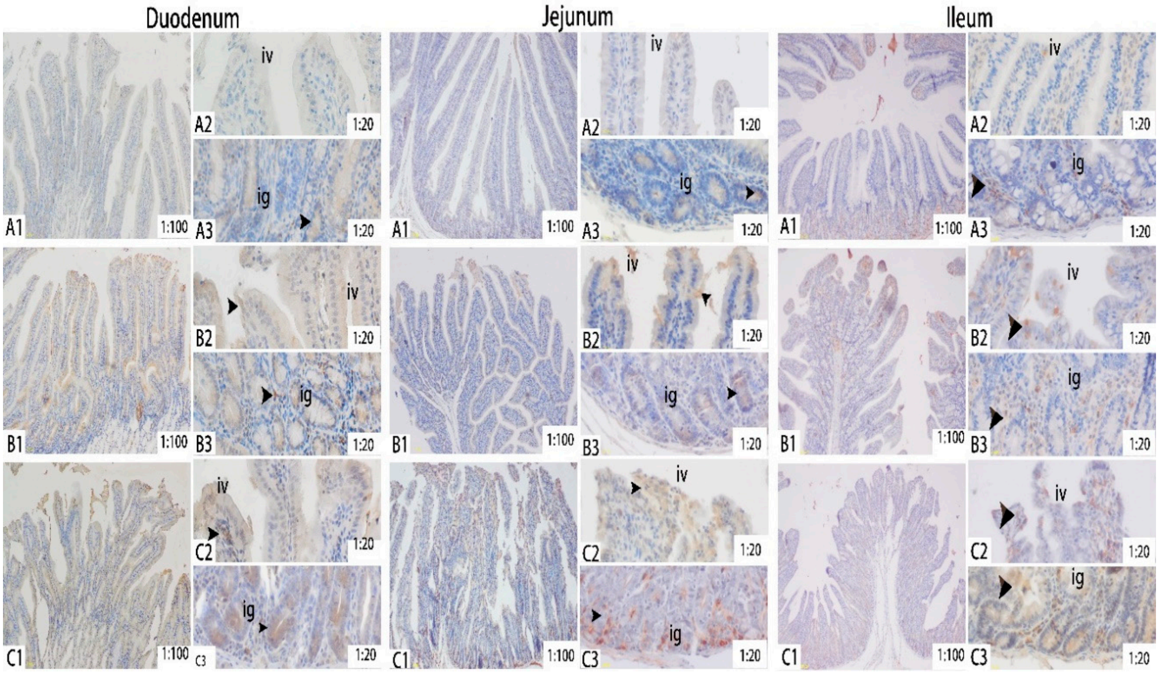

Figure S4. The distribution of IL-2 was tested via IHC method. Group A means the control group, groups B and C means added DON at 0.5 mg/kg BW and 1.5 mg/kg BW respectively. The 1:100 and 1:20 represent the magnification of electron microscopy is 100× and 400× respectively. The brown positive reactants was emphasized by arrow.

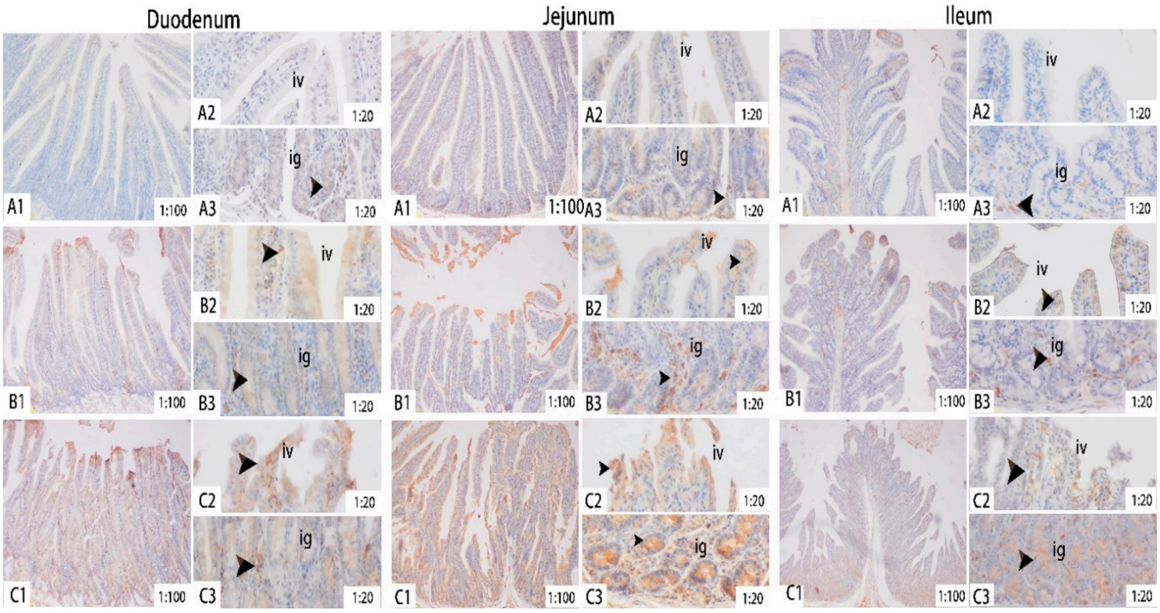

Figure S5. The distribution of IL-6 was tested via IHC method. Group A means the control group, while groups B and C means added DON at 0.5 mg/kg BW and 1.5 mg/kg BW respectively. The 1:100 and 1:20 represent the magnification of electron microscopy is 100× and 400× respectively. The brown positive reactants was emphasized by arrow.

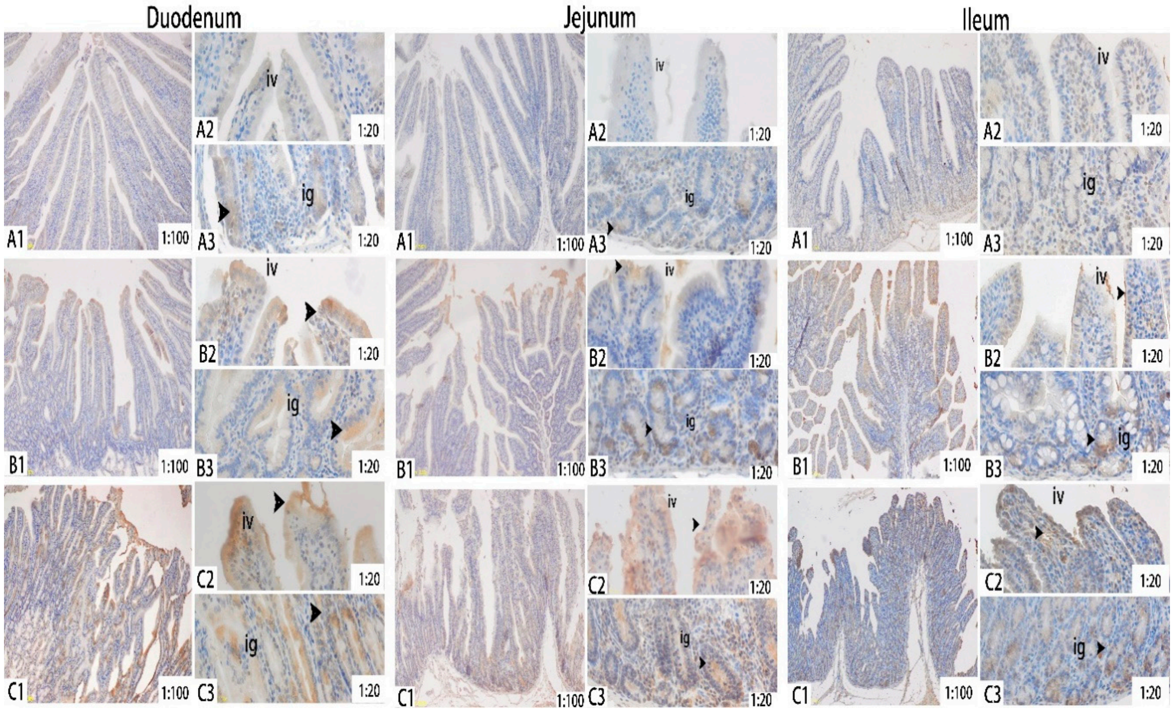

Figure S6. The distribution of IL-8 was tested via IHC method. Group A means the control group, while groups B and C means added DON at 0.5 mg/kg BW and 1.5 mg/kg BW respectively. The 1:100 and 1:20 represent the magnification of electron microscopy is 100× and 400× respectively. The brown positive reactants were emphasized by arrow.
